# Are there increased periprocedural complications with the MRI-conditional Medtronic Revo SureScan Pacing System? A meta-analysis

**DOI:** 10.1007/s12471-018-1086-4

**Published:** 2018-02-06

**Authors:** M. Shurrab, A. Kaoutskaia, A. Baranchuk, C. Lau, T. Singarajah, I. Lashevsky, D. Newman, J. S. Healey, E. Crystal

**Affiliations:** 1 Arrhythmia Services, Schulich Heart Centre, Sunnybrook Health Sciences Centre, University of Toronto, Toronto, Ontario Canada; 2 Division of Cardiology, Women’s College Hospital, University of Toronto, Toronto, Ontario Canada; 3 Institute of Health Policy, Management and Evaluation, University of Toronto, Toronto, Ontario Canada; 4 Division of Cardiology, Kingston General Hospital, Queen’s University, Kingston, Ontario Canada; 5 Population Health Research Institute, McMaster University, Hamilton, Ontario Canada

**Keywords:** Pacemakers, MRI, Complications, meta-analysis

## Abstract

**Background:**

The use of magnetic resonance imaging (MRI)-conditional permanent pacemakers has increased significantly. In this meta-analysis, we examine the safety of MRI-conditional pacing systems in comparison with conventional systems.

**Methods:**

An electronic search was performed using major databases, including studies that compared the outcomes of interest between patients receiving MRI-conditional pacemakers (MRI group) versus conventional pacemakers (control group).

**Results:**

Six studies (5 retrospective and 1 prospective non-randomised) involving 2,118 adult patients were identified. The MRI-conditional pacemakers, deployed in 969 patients, were all from a single manufacturer (Medtronic Pacing System with 5086 leads). The rate of pacemaker lead dislodgement (atrial and ventricular) was significantly higher in the MRI group (3% vs. 1%, OR 2.47 (95% CI 1.26; 4.83), *p* = 0.008). The MRI group had a significantly higher rate of pericardial complications (2% vs. 1%, OR 4.23 (95% CI 1.18; 15.10), *p* = 0.03) and a numerically higher overall complication rate in comparison with the conventional group (6% vs. 3%, OR 2.02 (95% CI 0.88; 4.66), *p* = 0.10) but this was not statistically significant.

**Conclusions:**

In this meta-analysis, the rates of pacemaker lead dislodgement and pericardial complications were significantly higher with the Medtronic MRI-conditional pacing system.

## Introduction

There is a worldwide steady and growing use of permanent pacemakers (PPMs); every year 600,000 PPMs are implanted and this number is increasing [1]. Interestingly, 50–75% of patients with a PPM may have an indication for MRI scanning during their lifetime, which urged the development of MRI-compatible PPM [2]. In 2011, the US Food and Drug Administration approved the first MRI-conditional pacing system in the United States: the Medtronic Revo SureScan Pacing System, the Generator and two CapSureFix MRI 5086 active-fixation pacing leads. To minimise the effects of the magnetic field, multiple design changes have been incorporated, which led to a stiffer lead that transfers more torque than other modern active fixation leads [3]. Many trials have confirmed the safety of performing MRI scanning on patients with these devices but very few looked at the safety of implantations and outcomes of the actual procedures [4, 5]. We thus conducted a meta-analysis to examine specifically the safety of MRI-conditional pacing systems in comparison with conventional systems.

## Methods

### Literature search and data sources

An electronic literature search was performed by two investigators (MS, AK) using PubMed until 28 August 2017. The search terms were: pacemaker AND magnetic resonance imaging. Neither language nor demographic restrictions were applied. All references from papers obtained through the database were reviewed manually. In addition, we performed a manual web search looking at different manufacturers’ databases as well as conference websites and proceedings. We only included full papers and excluded reports that did not provide full data about the outcomes of interest. The electronic search has been archived and is available upon request.

### Study selection and quality assessment

The inclusion was limited to studies which:

1. compared the outcomes of interest between patients receiving MRI-conditional PPMs (MRI group) versus conventional PPMs (control group);
2. included an adult population >18 years old; and
3. provided comprehensive data on outcomes of interest.

The selection of studies was assessed independently by three assessors (MS, AK, TS). We excluded non-comparative trials, crossover studies, case reports, editorials, letters, replies, and reviews.

### Data extraction

Three reviewers (MS, AK, TS) independently extracted the data from published sources; disagreements were resolved by discussion and, when necessary, in consultation with other co-authors.

Outcomes of interest were:

1. The rate of pacemaker lead dislodgement (atrial and ventricular);
2. pericardial complications (including pericarditis, pericardial effusion and cardiac tamponade);
3. overall complications; and
4. pacemaker parameters including sensing and pacing thresholds and impedance.

Whenever possible, direct communication with the authors of the papers was undertaken in an attempt to obtain the data of interest if presentation in the manuscript was incomplete.

We used the Newcastle-Ottawa Scale to further assess the quality of the observational studies. Studies were judged on three broad perspectives:

1. selection of the study groups;
2. comparability of the groups; and
3. ascertainment of either the exposure or outcomes of interest for case-control or cohort studies, respectively [6].

### Statistics

The software package RevMan (version 5), provided by the Cochrane Collaboration, was used for combining outcomes from the individual studies and statistical analysis. Outcomes were pooled using a random-effects model described by DerSimonian and Laird [7]. Summary estimates and 95% confidence intervals (CIs) were reported for dichotomous variables as odds ratio (OR). The heterogeneity between studies was assessed using Cochrane’s *X*^*2*^ and *I*^*2*^. An *I*^*2*^ > 50% was considered to represent significant heterogeneity [8]. Statistical significance was set at *p* < 0.05. We calculated the weighted means for the variable baseline characteristics and complication outcomes whenever possible. Otherwise we captured the medians with range or interquartile range as reported in the individual studies.

## Results

### Summary of the studies

**Fig. 1:**
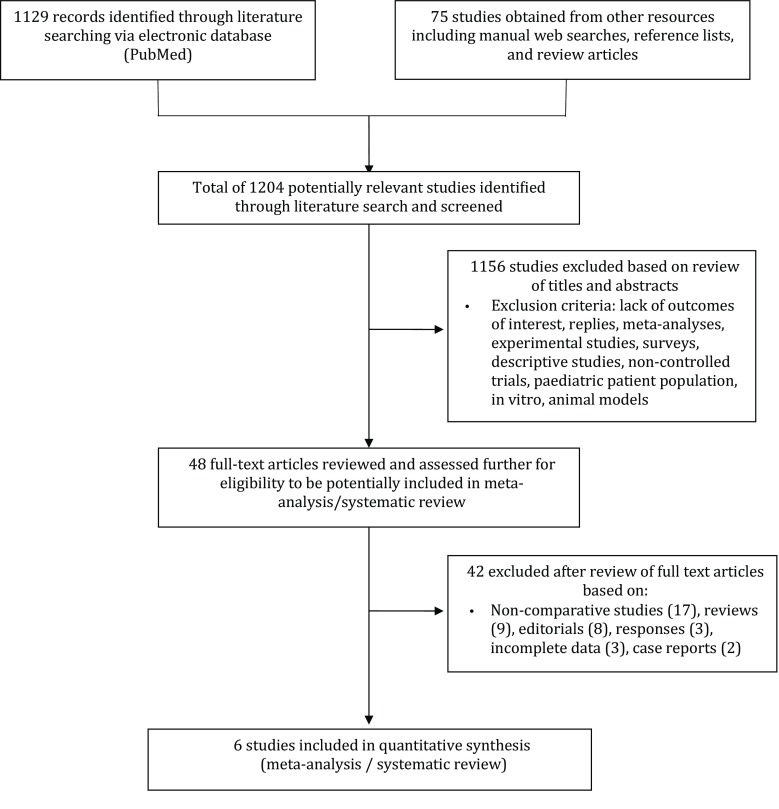
Flow diagram of literature search and study selection

The literature search resulted in 1,204 studies (1,129 from electronic databases and 75 from other resources including web searches and reference lists). We identified six studies (5 retrospective and 1 prospective non-randomised) that met all the inclusion criteria of this meta-analysis [3, 9–13]. The information relevant to the literature search is shown in Fig. 1. All studies that met our inclusion criteria used the Medtronic Revo SureScan Pacing System (with Medtronic CapSureFix 5086 leads) as the MRI-conditional pacing system. Different pacing systems were used in the control group. Tab. 1 presents a summary of the included studies, the pacing systems used, and the follow-up periods in the individual studies.

**Table 1:** Summary of included studies

Study/Year	Type of Study	No. of patients		Type of Lead		Follow-up
(*n* = 5)		MRI (*n* = 969)	Control (*n* = 1,149)	MRI	Control	(Months)
Forleo 2010 [10]	Prospective, non-randomised, controlled study	50	57	Medtronic CapSureFix 5086 MRI SureScan	Medtronic CapSureFix Novus 4076	12
Wollmann 2011 [12]	Retrospective study	39	59	Medtronic CapSureFix 5086 MRI SureScan	Medtronic 4592 (atrial leads); 4092 (ventricular leads)	6.5 ± 2.75^a^
Elmouchi 2014 [3]	Retrospective case-control study	65	92	Medtronic CapSureFix 5086 MRI SureScan	Medtronic CapSureFix Novus 5076	14
Rickard 2014 [11]	Retrospective cohort study	466	316	Medtronic CapSureFix 5086 MRI SureScan	Medtronic 5076 and Medtronic 4193 (left ventricular leads)	25.5^b^
						44.3^c^
Acha 2015 [9]	Retrospective, non-randomised, case-series study	72	420	Medtronic CapSureFix 5086 MRI SureScan	CapSureFix Novus 4076 and 5076	34
Kwon 2016 [13]	Retrospective study	277	205	Medtronic CapSureFix 5086 MRI SureScan	Medtronic CapSureFix Novus 5076	1

^a^Mean ± standard deviation

^b^Control group

^c^MRI group

### Baseline characteristics of patients

In total, 2,118 patients were included in this study. Medtronic MRI-conditional Revo SureScan Pacing Systems with 5086 leads (MRI group) were used in 969 patients. Baseline demographics and clinical characteristics were similar between the MRI and control group (age: 68 ± 2.4 vs. 72 ± 4.9 years, *p* = 0.11; male gender: 58% vs. 55%, *p* = 0.64; history of atrial arrhythmias: 39% vs. 29%, *p* = 0.43; hypertension: 55% vs. 62%, *p* = 0.97; and left ventricular ejection fraction: 60 ± 1.99 vs. 60 ± 3.70, *p* = 0.60). A summary of the baseline characteristics is presented in Tab. 2.

**Table 2:** Patient baseline characteristics

Variable	MRI	Control	*P*-value
Total patients (no.)	969	1,149	*n*/a
Age (years) (mean ± SD)	68 ± 2.43	72 ± 4.89	0.109
Male gender	58%	55%	0.635
History of atrial arrhythmias	39%	29%	0.432
Hypertension	55%	62%	0.967
Left ventricular ejection fraction	60 ± 1.99%	60 ± 3.70%	0.603

*n/a* not applicable

### Outcomes of interest

**Fig. 2:**
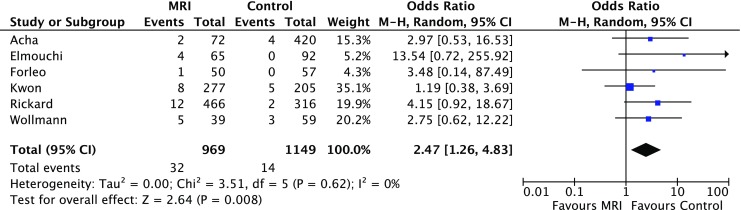
Forest plot of the individual and combined rates of atrial and ventricular lead dislodgements

The rate of pacemaker lead dislodgement (atrial and ventricular) was significantly higher in the MRI group (3% vs. 1%, OR 2.47 (95% CI 1.26; 4.83), *p* = 0.008). No significant heterogeneity was noted for this comparison (*I*^*2*^ = 0%, *p* = 0.62) (Fig. 2).

**Fig. 3:**
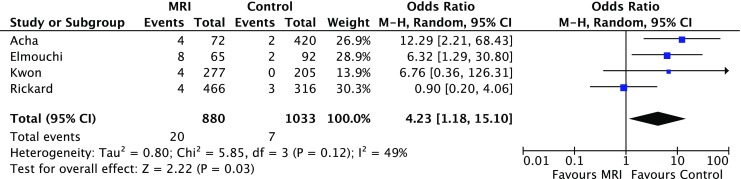
Forest plot of the individual and combined rates of pericardial complications

**Fig. 4:**
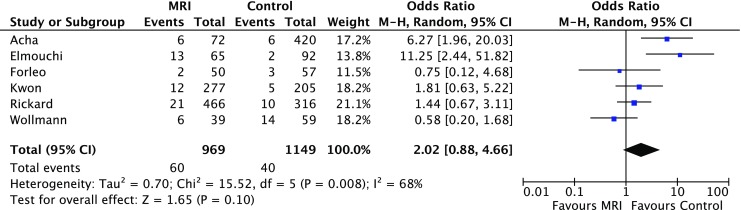
Forest plot of the individual and combined rates of overall complications

All studies reported the overall complication rate. The MRI group had a significantly higher rate of pericardial complications (2% vs. 1%, OR 4.23 (95% CI 1.18; 15.10), *p* = 0.03) and a numerically higher overall complication rate in comparison with the control group (6% vs. 3%, OR 2.02 (95% CI 0.88; 4.66), *p* = 0.10) but was not statistically significant, as shown in Figs. 3 and 4 respectively. Tab. 3 shows the rates of different individual complications.

**Table 3:** Comparison of outcomes

	MRI in %	Control in %	*P*-value
Total complications	6.19	3.48	0.100
Pericardial complications^a^	2.27	0.68	0.030
Total leads dislodgement	3.30	1.22	0.008

Mean calculated as weighted mean

^a^Pericardial complications: pericarditis, pericardial effusion, cardiac tamponade

Sensing parameters of atrial and ventricular leads immediately postoperatively and at follow-up (ranges between 6 weeks and 12 months) were significantly lower in the MRI group. In fact, ventricular lead pacing thresholds were significantly higher in the MRI group at follow up. Tab. 4 shows all the lead parameters postoperatively and at follow-up.

**Table 4:** Lead parameters

	Atrial leads			Ventricular leads		
	MRI	Control	*P*-value	MRI	Control	*P*-value
*Parameters at implant*						
– Sensing (mV)	2.71 ± 0.06	3.23 ± 0.06	0.00035	9.29 ± 1.04	13.41 ± 1.31	0.062
– Impedance (Ω)	562.94 ± 54.24	560.17 ± 47.13	0.171	703.34 ± 156.90	729.25 ± 105.31	0.621
– Pacing (V)	0.69 ± 0.16	0.64 ± 0.05	0.456	0.63 ± 0.11	0.59 ± 0.01	0.335
*Parameters at follow-up* ^a^						
– Sensing (mV)	2.84 ± 0.21	3.55 ± 0.15	0.007	9.94 ± 0.84	15.45 ± 1.50	0.012
– Impedance (Ω)	505.20 ± 14.11	514.64 ± 14.00	0.745	539.31 ± 12.49	619.74 ± 80.83	0.237
– Pacing (V)	0.76 ± 0.21	0.71 ± 0.21	0.935	0.92 ± 0.21	0.72 ± 0.21	0.038

Mean calculated as weighted mean ± standard deviation

^a^Follow-up range between 6 weeks and 12 months

## Discussion

Our meta-analysis has demonstrated the following main findings: The rate of pacemaker lead dislodgement (atrial and ventricular) was significantly higher with the use of Medtronic MRI-conditional Revo SureScan Pacing Systems with 5086 leads, with a significantly higher rate of pericardial complications and numerically higher overall complications in comparison with the conventional group.

Many reports have focused on the feasibility and safety of performing MRI scanning on patients with these devices but very few studies have looked at the safety and outcomes of the actual procedures [4, 5]. Although the studies included in our meta-analysis showed consistently higher complication rates, specifically the rate of pericardial complications with the Medtronic MRI-conditional Revo SureScan Pacing Systems with 5086 leads, two large randomised trials have shown a slightly lower (but likely not significant) rate of pericardial complications. In the EnRhythm MRI Study [14] and the Advisa MRI Study [15], there was 1–2% rate of perforation and/or pericardial effusion. Both studies represent specific clinical studies that follow unique, extensive protocols and implanting techniques. This includes pre-implant helix extension and retraction, using slower pin rotations, avoiding driving the lead with a fully seated stylet and avoiding the reverse ‘helicoptering’ of the ‘pinch-on’ tool once the helix is extended [3, 9]. Our meta-analysis represents rather more real-world procedural outcomes that reflect a wide variety of clinical practice.

Although the 5086 lead is a fairly new pacing lead, the outcomes of our study showing a lower safety profile for MRI-conditional PPMs are unlikely to be solely due to the learning curve of implanting physicians with this relatively new technology [16]. There are many specific design changes incorporated into the leads, including decreasing the number of filars and increasing diameter, increasing the number of turns in the inner coil and increasing the outer lead diameter [3]. As a result of these changes, the MRI lead is thicker, stiffer and transfers more torque. Sensing parameters of atrial and ventricular leads immediately postoperatively and at follow-up were significantly lower in the MRI group. Also, ventricular lead pacing thresholds were significantly higher in the MRI group at follow-up. While technically (statistically) true, it is unlikely that any of those differences would lead to any meaningful clinical outcome such as under sensing or battery longevity issues.

Three Medtronic leads (5076, 4074 and 4574) have been recently approved for use in the MRI environment and they are smaller in size in comparison with MRI-conditional leads with long-term implanting experience. Hence it is expected that these newly approved MRI-compatible pacing systems will have a wider usage and favourable outcomes, replacing the 5086 pacing leads in the near future. Moreover, other manufacturers have recently introduced MRI-conditional pacing systems; however, more reports are required to assess their safety.

### Limitations

Some studies were of limited quality given their retrospective nature and single-centre design. The results of our meta-analysis are based on the use of only a specific MRI-compatible lead, and cannot be generalised to all MRI-compatible systems. The discrepancy in follow-up periods among the studies could affect the outcomes. Assessing outcomes such as the complication rate is rather complex and multifactorial. Factors such as different levels of overall and system-specific experience among operators may alter our conclusions. The limited number of articles to be adopted, the small number of events and the short observation period are significant limitations of this meta-analysis. There could have been a lack of statistical power for some outcomes. Analysis related to some of the outcomes showed significant heterogeneity such as overall complications. Nevertheless outcomes such as lead dislodgements had insignificant heterogeneity that could reflect some similarities among studies.

## Conclusion

This meta-analysis supports the safety of conventional PPMs in comparison with the Medtronic MRI-conditional Revo SureScan Pacing Systems with 5086 leads. The rate of pacemaker lead dislodgement was significantly higher in the MRI group. In addition, there were significantly more pericardial complications and a numerically higher overall complication rate in comparison with the conventional group.

### Abstract presentation

Is there an increased complication rate with MRI-compatible PPM in comparison to conventional PPM? A meta-analysis. Heart Rhythm, Vol. 13, No. 5, May Supplement 2016.
